# Trending Weight Loss Between Usual Care and Bariatric Surgery Among Higher Weight Persons With Obstructive Sleep Apnea

**DOI:** 10.7759/cureus.32052

**Published:** 2022-11-30

**Authors:** Mariam Louis, Bijal Patel, Edward Prange, Brian Celso

**Affiliations:** 1 Pulmonology, University of Florida College of Medicine-Jacksonville, Jacksonville, USA; 2 Medicine, University of Florida College of Medicine-Jacksonville, Jacksonville, USA; 3 Surgery, University of Florida College of Medicine-Jacksonville, Jacksonville, USA

**Keywords:** weight reduction, bariatric surgery, health disparities, obstructive sleep apnea, obesity

## Abstract

Background

This study aimed to investigate the actual weight change documented as a goal of treatment after patients were newly diagnosed with obstructive sleep apnea (OSA). We hypothesized that patients with OSA and classified as overweight and obese based on BMI would fail to achieve significant weight loss over a two- to five-year period.

Methodology

This retrospective review included adults aged 18 years or older who were newly diagnosed with OSA in 2015, as indicated by a full nocturnal polysomnogram and using the 4% rule for the definition of hypopnea. Data collected were between January 01, 2015, and December 31, 2020. Patients received either usual care for weight reduction or bariatric surgery to assess the overall weight loss and identify barriers. Statistical analysis included independent t-tests, Mann-Whitney U tests and related samples McNemar change statistics, Cox proportional hazards regression, and Kaplan-Meier curves to analyze age, gender, ethnicity, and weight differences between usual care and bariatric surgery groups.

Results

The number of participants included for usual care and bariatric surgery was 100 and 24, respectively. Over five years, 87% of the usual care patients remained in the same BMI classification, 7% lowered their classification, and 6% raised theirs. For usual care patients, the average net weight per individual of 2.19 kg gained represented a 1.96% weight change. Bariatric patients lost an average net weight of 30.40 kg (22.39%). Cox proportional hazards regression showed that the overall model fit was statistically significant (χ^2^ = 55.40, degrees of freedom [df] = 9, and *P*-value < 0.001). The significant variables were time-dependent weight change and ethnicity. The Kaplan-Meier curve revealed that weight loss reduced over time in treatment.

Conclusions

This study confirmed that despite the direction to lose weight, only 7% of OSA patients lowered their BMI classification. Patient instruction and provider-driven weight loss strategies seem equally ineffective to achieve sustained weight reduction among high-risk groups. More research is needed to investigate optimal strategies that include interprofessional collaborative practices for sustained weight loss.

## Introduction

Obstructive sleep apnea (OSA) is the most common type of sleep disorder, affecting 10%-30% of adults in the United States [1]. Obesity, a major risk factor for OSA, was estimated to be as high as 45% in individuals with OSA, and about 70% of adult patients with OSA have obesity [2]. Excess body weight has been hypothesized to impact breathing by altering upper airway structure and function and disturbing the relationship between respiratory drive and load compensation [3]. In addition, higher weight appears to exacerbate OSA events via obesity-related reductions in residual capacity and increased whole-body oxygen demand.

The American Thoracic Society (ATS) guideline for weight reduction through lifestyle modification (i.e., healthy eating, physical activity, and eating behavior modification) seems to improve related disturbances of metabolic syndrome and remains a cornerstone intervention for overweight patients with OSA [4]. The ATS guideline emphasizes that an essential component of OSA treatment was the early prevention of further weight gain to maintain a healthy body weight in patients with OSA. Likewise, the American Academy of Sleep Medicine (AASM) has published several practice parameters on various therapies available for successful dietary weight loss shown to enhance the management of OSA [5]. Therefore, the AASM also recommended dietary weight loss for all patients with OSA.

Past research found that diet, exercise, and behavioral counseling resulted in only 5%-10% average weight loss [6]. For many patients with OSA, the achieved weight loss tends to be short-lived, and a few individuals were able to maintain an ideal body weight over time [7,8]. This suggests that behavioral weight loss strategies are less than beneficial in achieving sustained weight loss for patients with OSA. The limited weight loss maintained through conventional weight loss programs has promoted the use of weight loss surgery for severe obesity. Studies that assessed surgical weight loss interventions in patients with OSA found an average loss of 13.5% of initial body weight over one year [2]. Moreover, Gloy et al. [9] found the weight loss option of bariatric/metabolic surgery to be more effective at treating obesity, diabetes, and metabolic syndrome.

Current OSA practice guidelines recommend clinicians emphasize weight loss through diet and exercise to improve health. This study aimed to investigate the actual weight change documented as a goal of treatment after patients were newly diagnosed with OSA. We hypothesized that patients with OSA who were classified as overweight and obese based on BMI would not achieve significant weight loss over a two- to five-year period. The secondary outcomes were to examine the minority status of higher weight patients with OSA and compare the patients directed to lose weight with those who underwent bariatric surgery over the same study period. This paper was previously presented as an oral presentation at the 2021 CHEST Annual Meeting on October 20, 2021.

## Materials and methods

Participants

This study included 124 new patients (100 usual care and 24 bariatric surgery) who were evaluated and newly diagnosed with OSA at a sleep center in Jacksonville, Florida, between January 01, 2015, and December 31, 2015. A recommendation for weight loss through dieting and exercise was established as treatment goals for newly diagnosed patients with OSA.

Inclusion Criteria

Patients aged 18 years or older and newly diagnosed with OSA as indicated either by the International Classification of Diseases (ICD) 9 or ICD 10 and received usual weight loss practices were included in this study. The diagnosis and severity of OSA were established by a full nocturnal polysomnogram and defined by the cutoffs for the apnea-hypopnea index recommended by the American Academy of Sleep Medicine, using the 4% rule for the definition of hypopnea. A comparator group consisted of patients who chose weight loss through surgical intervention during the same study period. To perform all statistical calculations, both groups were required to have a complete medical record (including weight and BMI classification).

Exclusion Criteria

Pregnant females, patients with severe mental disorders (e.g., schizophrenia, schizoaffective, and bipolar disorder), patients on prescribed psychiatric medications associated with weight gain, patients with a history of a substance use disorder (i.e., alcohol, marijuana, cocaine, and opioids), patients on long-term steroid therapy, and patients with insufficient medical records requiring to determine a BMI classification.

Study design

The University of Florida Institutional Review Board (IRB202100540) obtained the study's approval. First, potential participants who met inclusion criteria were obtained from a search performed by the integrated data repository Epic electronic health records (EHRs; Epic Systems Corporation, Verona, WI, USA) for examination. The search query was based on patients who received either the ICD-9-CM Diagnosis Code for OSA of 327.23 or the ICD-10-CM Diagnostic Code G47.33 for patients with a new diagnosis of OSA in 2015. Patient consent was waived by the IRB as no direct patient contact was required to complete the study. The medical records of participants initially treated at the UF Health Pulmonology Jacksonville Sleep Disorder Clinic between January 01, 2015, and December 31, 2015, were reviewed for the next two to five years, depending upon the length of their treatment before being discharged from the clinic.

This was a retrospective chart review of patients with both an OSA diagnosis and a recorded weight and BMI classification. Data was collected between January 01, 2015, and December 31, 2020. The comorbidities recorded included asthma, chronic obstructive pulmonary disease, diabetes mellitus, heart disease, hypertension, renal disease, rheumatoid arthritis, and thyroid. The types of prescribed medications noted included antidepressants, antihyperglycemics, antihypertensives, corticosteroids, and oral contraceptives. The data collected was entered and stored exclusively on the Research Electronic Data Capture (REDCap, Nashville, TN, USA) research site and then deidentified at the end of the study. Medical information was obtained from Epic EHR, Epic Systems Corporation.

Statistical analysis

The statistical approach used to analyze the data collected first aggregated and quantified the demographic information such as age, gender, ethnic group, and BMI classification. Statistical analysis consisted of the average age and percentages of gender, ethnic group, comorbidities, and prescribed medications. The t-test was used to compare the age, initial and final weights, and initial and final BMIs of the instructed male and female participants. The Mann-Whitney U test was used to compare the usual care group with the bariatric surgery group. Related samples McNemar change statistics were used to construct a 2 × 2 classification table. We analyzed the difference between the paired proportions (recorded BMI at the initial assessment with the final recorded BMI classification). McNemar's test was conducted to determine if a statistically significant difference existed between the classification proportions (expressed as a percentage) over the two to five years during which participants received treatment for OSA.

A Cox proportional hazards regression was performed to assess the magnitude of relationships between the predictor variables: age, ethnic group, number of comorbidities, medications, and time in treatment with BMI classification (based on their initial weight). Normal/underweight versus overweight/obese was the hazardous event of weight change at the end of the study period. The Kaplan-Meier method was used to calculate survivor curves that measured the overall weight loss over time for comparison between the usual care and bariatric surgery groups. Weight in kilograms was squared to eliminate negative values. Significance was determined using the alpha level of 0.05. All statistics were calculated using IBM Corp. Released 2021, IBM SPSS Statistics for Windows (Version 28.0, Armonk, NY, USA).

## Results

The total sample size for the retrospective chart review was 124 consisting of 100 OSA patients (36 men and 64 women) directed to lose weight and 24 bariatric/metabolic surgery patients (5 men and 19 women). The percentages of participants’ comorbidities were asthma (28.8%), chronic obstructive pulmonary disease (25.8%), diabetes mellitus (37.9%), heart disease (16.7%), hypertension (78.8%), renal disease (0%), rheumatoid arthritis (3%), and thyroid (15.2%). The percentages of weight-associated medications prescribed to participants were antidepressants (42.4%), antihyperglycemics (45.8%), antihypertensives (81.4%), corticosteroids (3.4%), and oral contraceptives (0.0%).

Table 1 shows the participants' characteristics (in percentages) in the usual care and bariatric groups for ethnicity, BMI classification, comorbidities mode (number), and known medications mode (number) that potentially increased weight.

**Table 1:** Participants' characteristics. BMI, body mass index

	Usual care	Bariatric surgery
Gender (Female)	64 (64%)	19 (79%)
Ethnicity		
African American	45%	37%
Asian	1%	0%
Caucasian	52%	63%
Hispanic	1%	0%
Other	1%	0%
BMI classification		
Underweight	0%	0%
Normal	10%	4%
Overweight	9%	25%
Obese	81%	71%
Comorbidities mode	2	1
Medications mode	2	0

The t-test results for the variables age, initial weight and final weight, and initial and final BMIs of the female participants compared with the male participants were not statistically significant (Table 2).

**Table 2:** Results of t-tests for female and male patients with OSA. *Nonsignificant. BMI, body mass index; OSA, obstructive sleep apnea

	Female	Male	*P*-value
Age (in years)	55.02 (11.48)	52.58 (13.72)	0.346^*^
Initial weight (kg)	106.87 (33.08)	121.12 (37.00)	0.051^*^
Final weight (kg)	114.83 (35.93)	109.01 (36.27)	0.440^*^
Initial BMI	40.17 (11.87)	36.96 (11.49)	0.193^*^
Final BMI	40.88 (12.16)	37.55 (12.59)	0.197^*^

The Mann-Whitney U test results for the variables age, initial weight and final weight, and initial and final BMIs of the usual care group compared with the bariatric surgery group showed that the age, initial weight, and initial BMI were statistically significant (Table 3).

**Table 3:** Mann-Whitney U tests for directed (usual care) versus bariatric surgery patients. BMI, body mass index

	Usual care	Bariatric surgery	P-value
Age (years)	56.69 (12.41)	44.35 (11.82)	<0.001
Initial weight (kg)	111.67 (34.70)	135.78 (26.05)	<0.001
Final weight (kg)	112.74 (35.98)	103.03 (29.35)	0.234
Initial BMI	39.01 (11.78)	48.55 (8.93)	<0.001
Final BMI	39.57 (12.35)	37.09 (10.68)	0.383

The related samples McNemar change statistics that exhibited the trend in weight change over five years for both groups were not statistically significant (McNemar = 3.125, *P*-value = 0.070).

Independent predictors of higher weight

A total of 7% of the usual care participants directed to lose weight lowered their BMI classification, while 6% raised their BMI classification. The majority of usual care patients (87%) remained in the same BMI classification over the five years of the study. An average net amount of weight per individual of 2.19 kg gained represented a 1.96% weight increase over the study period. In comparison, while 29.2% of participants lowered their BMI classification, none of the participants in the bariatric/metabolic surgery group raised their BMI classification. Notably, 70.8% of the bariatric surgery patients remained in the same BMI classification over the five years. The bariatric surgery patients lost an average net amount of 30.40 kg per individual, which represented a 22.39% weight change over the five-year study. A Cox proportional hazards regression for the directed group with the time-dependent weight variable calculated over five years and stratified by BMI classification and gender showed the overall model fit was statistically significant (χ^2^ = 55.40, degrees of freedom [df] = 9, and *P*-value < 0.001). Table 4 shows that the significant variables in the model were time-dependent weight change and ethnicity. 

**Table 4:** Cox proportional hazard regression results. ^*^Significant at 0.05. ^**^Significant at 0.01. ^†^Referenced to other.

Variables in the Equation	Beta	Standard error	Significance	Odds ratio
Time-dependent weight	2.993	1.449	0.039^*^	19.94
Age	0.018	0.015	0.247	1.018
Ethnicity^†^				
African-American	4.218	1.626	0.009^**^	67.90
Asian	0.316	0.267	0.236	1.372
Caucasian	0.427	1.228	0.728	1.532
Hispanic	3.945	1.468	0.007^**^	51.70
Comorbid	–0.207	0.131	0.113	0.813
Medication	0.059	0.174	0.735	1.061
Time in treatment	<0.001	<0.001	0.286	1.000

Time-dependent weight loss

**Figure 1:**
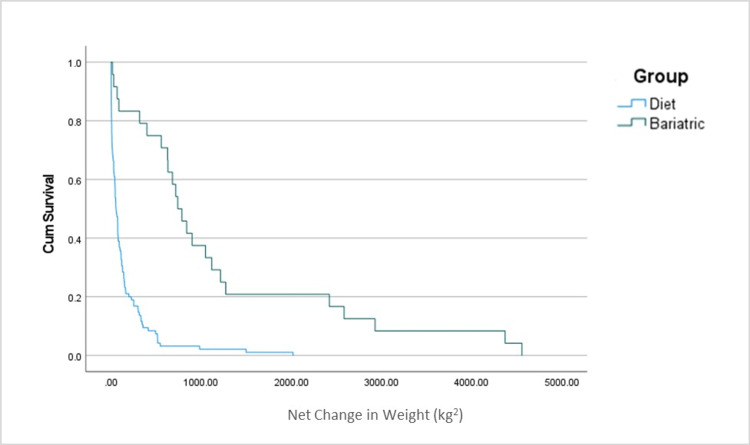
Survival functions for usual care versus bariatric surgery.

For the time-dependent weight loss variable, a participant who received usual care for weight loss was found to be nearly 20 times more likely to have an initial weight classification of overweight or obese across the five years of the study period while controlling for age, ethnic group, number of comorbidities, medications, and time in treatment. In addition, for the variable ethnicity, African-American and Hispanic participants were approximately 68 and 52 times more likely to have an initial weight classification of overweight or obese, respectively, controlling for all other variables. Table 4 shows the coefficients for the predictor variables in the model. Kaplan-Meier survivor curves for pairwise comparisons between the usual care and bariatric groups were statistically significant (Mantel-Cox = 37.74 and *P*-value = < 0.001). Figure 1 shows the survival curves that revealed bariatric surgery patients lost significantly more weight over time than the patients (usual care) directed to lose weight. However, weight loss for both groups dropped off substantially before OSA treatment ended.

## Discussion

The purpose of this study was to investigate the actual weight change documented as a goal of treatment after patients were newly diagnosed with OSA. This research confirmed that despite directions given by providers, the majority of patients (87%) maintained their BMI classification, whereas only 7% of the patients lowered their BMI classification. Moreover, 6% raised their BMI classification from either normal to overweight or overweight to obese. This suggests that instruction on weight reduction through lifestyle modification alone such as diet and exercise was ineffective. Additionally, the study indicated that weight loss was highest within the first 6 to 12 months. It appears that while patients with higher weight diagnosed with a sleep disorder were counseled about weight, the weight that was lost occurred early on only to decrease sharply over time. Nevertheless, the length of time a patient was in treatment for OSA did increase the odds a patient might lose weight by approximately 20-fold.

Our results coincide with previously published weight reduction studies. In particular, Hoyt and Walter [10] found that initial BMI was the best predictor of subsequent BMIs across time. A randomized controlled trial followed diabetic patients with OSA on dietary weight loss for 10 years [11]. Investigators compared an intensive weight loss program against diabetes support and education. The intensive group had significant weight loss reduction compared with the diabetes education and support group. Similarly, in a meta-analysis, Mitchell et al. [12] showed that a more intense behavioral therapy resulted in more weight loss. However, there did not appear to be a threshold amount of weight loss needed to improve OSA severity, in that more weight loss was associated with greater benefit [13]. In contrast to our findings, Gottlieb and Punjabi [14] found no significant association between the duration of intervention and study effect size for BMI outcome.

The results of this study also showed that the odds for African-Americans and Hispanics were significantly more likely to have an initial higher weight. Wong et al. [15] found that racial differences existed in the decision to select laparoscopic sleeve gastrectomy and laparoscopic Roux-en-Y gastric bypass. The investigators showed that African-American and Hispanic patients were more likely to undergo laparoscopic sleeve gastrectomy than White, non-Hispanic patients. In addition, this association was significant regardless of preoperative BMI and sex for African-Americans. While it may seem that bariatric surgery could provide an advantage over counseling as usual care for weight loss, readmission rates after bariatric surgery were also shown to vary by race/ethnicity [16]. African-American and Hispanic patients had higher complication-related readmissions compared with White patients.

Age may also play a role in the selection of weight reduction techniques as younger patients with OSA and higher weight were more likely to undergo bariatric surgery. The current evidence implies that weight loss surgery is a more effective treatment option for weight reduction [17]. Similar research has shown that bariatric/metabolic surgery was associated with substantially lower mortality rates for all causes and longer life expectancy among adults with severe obesity [18]. Those patients with OSA who were younger and of higher initial weight were more likely to undergo bariatric surgery instead for weight loss. Although these individuals proved to be significantly more successful at weight loss, the weight lost was also greatest in the first 6 to 12 months of the study period similar to the group who received direction to lose weight alone.

This retrospective study illustrates that weight loss following bariatric surgery, when followed over time, tends to decrease. Past research showed that gastric bypass had the greatest reduction in weight and improvement in obesity-related comorbidities followed by sleeve gastrectomy than banding procedures [17,18]. Additionally, the surgical intervention led to reduced use of antidiabetic, antihypertensive, and lipid-lowering medications and improved quality of life. Wharton et al. [19] found that while improvements in health conditions such as glycemic control were established, the promise of dramatic weight reduction sustained over time had proved less than favorable. Significant weight regains (defined as >15% gain of initial weight loss post bariatric surgery) occurred in 25% to 35% of patients within 2 to 5 years following surgery and ranged from 25% to >70% after 10 years [20,21].

Health disparities

Ramos et al. [22] showed that the prevalence of OSA was greater among minorities. Although there are a few population-based studies, a significant amount of racial/ethnic minorities reported OSA-like symptoms. If left undetected, OSA may increase the risk for other health problems such as hypertension, diabetes mellitus, obesity, and stroke through its associations with potent vascular risk. Social determinants of health hazards disproportionally seen within minority groups living in disadvantaged neighborhoods have higher rates of poverty and unemployment and lack of secure and stable housing [23]. Likewise, higher readmission rates among minorities due to complications after bariatric surgery may be the result of poor access to health care and other health disparities in their communities.

The social stigma attached to obesity has been associated with psychological stress experienced by higher weight people. Evidence suggests that individuals with excess weight elicit negative feelings such as anger, disgust, and blame [24]. Higher weight people frequently find themselves the targets of derogatory comments, dislike, and prejudice from others. People with obesity do not always feel welcome by health care providers either. Healthcare professionals were shown to hold negative attitudes about individuals because of their excess body weight [25]. There is also an association between the type of weight reduction surgery, insurance status, cost of procedures, and socioeconomic status. The effect may be avoidance or postponement of needed health care that results in more advanced and difficult-to-treat conditions [15]. Comparable hospitals that serve as the safety net for the urban core and whose patients suffer significantly from social disadvantage may experience similar health disparities.

Clinical implications

The prompt recognition, diagnosis, and treatment of OSA are critical to decrease the associated morbidity and mortality as well as improve health-related quality of life through the outpatient management and therapeutic intervention of OSA [26]. There are several risk factors and related comorbidities implicated in the development and progression of OSA. Pien et al. [27] listed nonmodifiable risk factors to include advanced age, male gender, menopause, genetic disorders, and African American and Asian races. The modifiable risk factors included obesity, craniofacial/upper airway abnormalities, alcohol use, cigarette smoking, endocrine disorders (e.g., hypothyroidism), and the use of sedating medications. Undiagnosed and untreated OSA, however, was shown to be independently associated with hypertension, type II diabetes mellitus, stroke, daytime sleepiness, motor vehicle crashes, depression/mood disorders, and neurocognitive defects [17,28].

Providers treating OSA may be unfamiliar with evidence-based methods for achieving weight loss. The application of lifestyle modification recommendations into routine OSA care should be further investigated as sleep centers are not ordinarily designed to also accommodate weight management in clinical practice. Equally important is for providers to acquaint themselves with ethnic/cultural-appropriate levels of patient education and strategies on weight reduction processes suggested to vulnerable populations, including racial minorities. Therefore, achieving long-term success will likely require the implementation of interprofessional collaborative practice and new advances in technologies to become more effective [29,30]. For example, a nutritionist, physical therapist or personal trainer, and mental health practitioner trained in best practices for weight loss are invaluable additions to a treatment team to ensure patients’ healthier weights are sustained.

Study limitations

As the majority of weight loss typically occurred within the first year, the data was skewed, which violated the normality assumption and mostly limited the types of statistical procedures conducted to nonparametric tests. Another limitation of the study was the lack of adjustment for important confounding factors such as the severity of preexisting comorbidities and their relationships with any weight loss due to illness. The retrospective nature of this research did not allow for a follow-up OSA evaluation at the end of the study period. Therefore, it was not [possible to conclude any improvement in OSA symptoms. In addition, only a small minority underwent weight loss surgery. Despite the five-year duration of this study, the medical records of bariatric surgery participants were not reviewed to determine if any comorbidity was improved or in remission. Furthermore, medications associated with weight gain were not considered to determine if their use was reduced or discontinued after surgery. Finally, the results were not able to generalize beyond the participants who were exclusively from one institute in Northeast Florida.

## Conclusions

Obesity is a complicated disease, which is regarded as a life-long, progressive, genetically related, multifactorial disease due to excess fat storage. Our research suggests that further investigation is needed to determine an optimal strategy that facilitates weight reduction in patients with both OSA and higher weight. Moreover, disproportionate increases in the risk of obesity seen among minorities who are already among higher risk groups advocate for exploration into the role of social determinants of health and healthcare disparities. A patient-centered, shared decision-making strategy to select weight reduction techniques may benefit from the utilization of ancillary services. Thus, a multifaceted and interprofessional collaborative practice includes advanced weight loss training for medical professionals and more involved clinic visits to assist patients to overcome physical, social, and psychological barriers that currently hinder successful and sustained weight loss. For instance, providers may adopt a weight-neutral approach, which is a paradigm shift that takes the focus off weight and places it on health. In this way, it has positive health benefits to care adequately for a growing population of patients with OSA and obesity.
